# Enhanced anticancer potency with reduced nephrotoxicity of newly synthesized platin-based complexes compared with cisplatin

**DOI:** 10.1038/s41598-022-11904-3

**Published:** 2022-05-18

**Authors:** Roya Salehi, Selda Abyar, Fatemeh Ramazani, Ali Akbar Khandar, Seyed Abolfazl Hosseini-Yazdi, Jonathan M. White, Mahdi Edalati, Houman Kahroba, Mehdi Talebi

**Affiliations:** 1 Drug Applied Research Center and Department of Medical Nanotechnology, Faculty of Advanced Medical Sciences, Tabriz University of Medical Sciences, 5165665811 Tabriz, Iran; 2 Department of Inorganic Chemistry, Faculty of Chemistry, University of Tabriz, 5166614766 Tabriz, Iran; 3 Department of Molecular Medicine, Faculty of Advanced Medical Sciences, Tabriz University of Medical Sciences, Tabriz, Iran; 4 School of Chemistry and BIO-21 Institute, University of Melbourne, Parkville, Vic. 3010 Australia; 5 Department of Laboratory Sciences, Paramedical Faculty, Tabriz University of Medical Sciences, Tabriz, Iran; 6 Department of Toxicogenomics, GROW School for Oncology and Department Biology, Maastricht University, Maastricht, The Netherlands; 7 Center for Environmental Science, Hasselt University, Hasselt, Belgium; 8 Department of Applied Cell Science, Faculty of Advanced Medical Sciences, Tabriz University of Medical Sciences, Tabriz, 5154853431 Iran

**Keywords:** Biochemistry, Cancer, Chemical biology, Chemistry

## Abstract

As a platinum-containing anticancer drug, cisplatin is the keystone for treating many malignancies. Nephrotoxicity is the main dose-limiting toxicity, and several hydration therapies and supplementary strategies are utilized to reduce cisplatin-induced kidney damage, so the discovery and development of effective and safe antitumor drugs are still on the path of human health. Herein, a new four-coordinated Pt complex [Pt(TSC)Cl] using N(4)-phenyl-2-formylpyridine thiosemicarbazone (HTSC) was synthesized and characterized by single-crystal X-ray diffraction, ^1^HNMR, FT-IR, LC/MS and CHN elemental analysis. The Pt(TSC)Cl complex revealed antiproliferative activity against A549, MCF-7 and Caco-2 cell lines with a low micromolar IC_50_ (200–1.75 µM). Specifically, the Pt(TSC)Cl complex displayed more selectivity in Caco-2 cells (IC_50_ = 2.3 µM) than cisplatin (IC_50_ = 107 µM) after 48 h of treatment. Moreover, compared with cisplatin, a known nephrotoxic drug, the Pt(TSC)Cl complex exhibited lower nephrotoxicity against Hek293 normal cells. We also found that the Pt(TSC)Cl complex can effectively prevent cancer cell propagation in sub-G1 and S phases and induce apoptosis (more than 90%). Real time PCR and western analysis demonstrated that the expression pattern of apoptotic genes and proteins is according to the intrinsic apoptosis pathway through the Bax/Bcl-2-Casp9-Casp3/Casp7 axis. Collectively, our findings indicated that the Pt(TSC)Cl complex triggers apoptosis in Caco-2 cell lines, while low nephrotoxicity was shown and may be considered a useful anticancer drug candidate for colorectal cancers for further optimization and growth.

## Introduction

Based on World Cancer Statistics, as the third most prevalent cancer in Iran, colorectal cancer was responsible for 9% of all cancers in 2018. In the same year, colon cancer in Iran was classified fourth in view of occurrence and fifth in terms of mortality rate^1^. The frequency of colorectal cancer has risen during the past three decades in Iran^2^. The maximum prevalence of colorectal cancer was reported in the central, northern and western parts of the country^1,3^. In the class of metal-based complexes, cisplatin as a platinum (II)-based antitumor agent, has antineoplastic properties and has served in the treatment of several solid organ cancers, including colon, lung, testis, ovary, and breast cancers. Although toxicities involve ototoxicity, gastrotoxicity, myelosuppression, and allergic reactions^4^, the main dose-limiting adverse outcome of cisplatin is nephrotoxicity^5,6^. On the other hand, the efficiency of cisplatin is also restricted due to its general toxicity and resistance of tumors to this agent^7^. To overcome the restrictions of the current platinum drugs and to produce less toxic, more effective and selective anticancer agents, the linkage of other metal complexes with DNA has drawn the attention of researchers in medicinal chemistry^8,9^. Therefore, there is an urgent need to produce newer, more adaptable newer chemotherapeutic agents. Unceasing exploration for anticancer drugs with less toxicity and high efficacy is a significant orientation in anticancer drug studies^10,11^.

Thiosemicarbazone (TSC), as a pharmacophore, has attracted considerable attention from scientists due to its favorable biological properties, including overcoming multidrug resistance, antituberculosis, antiviral, antifungal, antimalarial, and, most interestingly, antineoplastic activity^11–15^. The anticancer activity of TSCs has arisen from many factors, including suppression of tumor cell aggression and migration^16^, chelating metal properties^17^, hindering DNA synthesis, ROS production^18^, and influence of numerous key proteins^19^. To date, various TSC-linked drug candidates have entered clinical trials. For example, 3-AP, a metal chelating agent, is presently in a phase II clinical trial^10^. COTI-2 and DPC^10^, another TSC-containing drug, enhanced the effectiveness and selectivity against cancer cells in comparison with 3-AP^12,17,20^. Despite numerous reports of this family of compounds, TSCs with intense cytotoxicity and great selectivity toward cancer cells and less adverse effects continue to be uncovered. Recently, it has been reported that a collection of TSC derivatives, such as 5n (TS-1), displayed significant antitumor activity and could block the migration of MGC803 cells to some degree^21^. Serda et al. has also reported potential anticancer activity and considerable cellular membrane permeability of thiosemicarbazone derivatives for pharmaceutical applications, showing that theses organic ligands play an important role in drug delivery/distribution^22^. Recently, with regard to malignant diseases, a series of novel thiosemicarbazone derivatives was intensively developed in multiple clinical phase I/II trials^12^. In another study, Veronika F.S.Pape and coworkers develop Thiosemicarbazone derivatives that display significant antitumor activity in multidrug resistant cancer cell lines overexpressing the drug efflux pump P-glycoprotein^11^. Harpreet Kaur et al. synthesized several Thiosemicarbazone derivatives with anticancer properties on different cancer cell lines^23^. Based on the role of TSC derivatives in hindering the growth of cancer cells and following previous works on the identification of novel TSC compounds, we describe the design and synthesis of a novel organic compound, N(4)-phenyl-2-formylpyridine thiosemicarbazone, and its new complex with platinum as well as the anticancer effects of this new complex.

## Materials and methods

All chemicals and reagents were obtained from Sigma Aldrich and used without purification.

FT-IR spectra were recorded on a Bruker Tensor 27 FT-IR spectrophotometer with KBr disks in the range 4000–400 cm^−1^ (Columbia, MD, USA). A QTOF LC/MS spectrometer was used for mass spectrometry (Billerica, MA, USA). 1HNMR spectroscopy was performed at room temperature on an NMR Bruker Advance 400 MHz magnet (Agilent, USA) using DMSO-d6. Elemental analysis, C H N, was performed by an elemental analyzer Vario EL III (Sydney, Australia). X-ray crystallography was performed on an Xtal AB Synergy Dualflex Hypix diffractometer using CuKα radiation, λ = 1.54184 Å at 100 K. The structure was solved by direct methods and different Fourier syntheses^24^. A thermal ellipsoid plot was generated using the Mercury program^25^ integrated within the WINGX^26^ suite of programs.

A Facs Canto II (Bio–Rad) flow cytometry system (Mexico, USA) with the Flow LogicTM program was used for cell cycle Annexin V and PI evaluation. Real time PCR test was performed by Mic real time PCR, Bio Molecular system (Santa Clara, USA).

### Synthesis and structural characterization of the complex

**Scheme 1:**
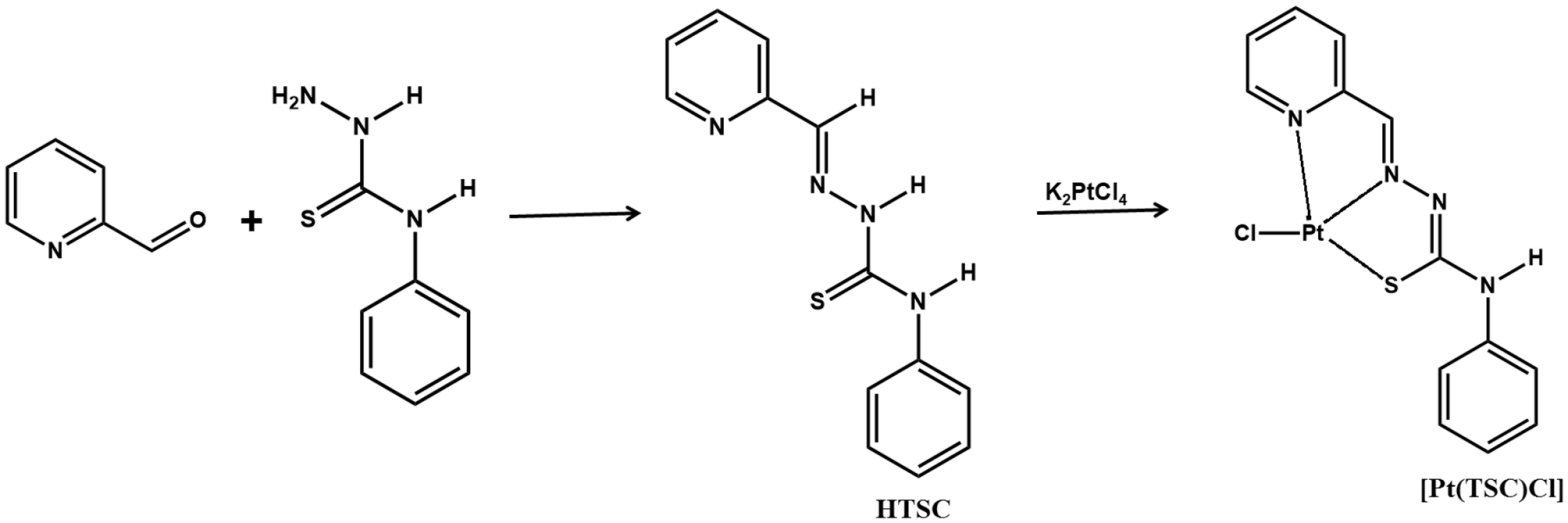
Synthetic root of the HTSC ligand and Pt(TSC)Cl complex.

The organic ligand (HTSC = N(4)-phenyl-2-formylpyridine) was prepared according to a literature procedure^27^ (Scheme 1). The Pt complex, [Pt(TSC)Cl], was synthesized by dropwise addition of aqueous solution (3 mL) of K_2_PtCl_4_ (0.206 g, 0.5 mmol) to 15 mL methanolic solution of HTSC (0.128 g, 0.5 mmol). The reaction mixture was stirred for three days at 25 °C. Upon evaporation of the solvent over 5 days, fine brown crystals suitable for single crystal X-ray crystallography were observed. The yield was 0.16 (65%, based on metal), Anal. Calc. (%) for C13H11ClN4PtS (formula weight = 485.86): C32.14, H 2.28, N 11.53, found (%) C 32.09, H 2.21, N 11.48. The obtained melting point for the Pt(TSC)Cl complex was 301 °C. Selected FT-IR data (KBr disk, cm-1):

### Cell culture and reagents

Human colon (Caco-2), lung (A549) and breast cancer cells (MCF-7) and embryonic kidney cells (Hek293), RPMI 1640 medium (Gibco) and fetal bovine serum (FBS) were obtained from the Pasteur Institute of Iran. MTT dye [3-(4,5-dimethyl thiazol-2-yl)-2,5-diphenyl tetrazolium bromide] was purchased from Alfa Aesar, Thermo Fisher Scientific, Heysham, UK. Penicillin–streptomycin (Pen-Strep, 100x) was purchased from Serana Europe GmbH, Germany. PI and RNase A were acquired from Sigma Aldrich. The Annexin V-FITC/PI Apoptosis Detection Kit was purchased from XBioscience Inc. The Western blotting antibodies consisted of Bax, Bcl-2, P27, pro-caspase3, cleaved caspase 3, pro-caspase7, cleaved caspase 7, pro-caspase9, cleaved caspase 9, and GAPDH.

### Cell viability assay

All cancer cells were cultured in RPMI 1640 medium with 10% FBS in a humidified incubator of 95% air and 5% CO2 at 37 °C. The IC_50_ values of platinum (II) complex, Pt(TSC)Cl, as an anticancer drug, and cisplatin on three different cancer cell lines, MFC-7, A549 and Caco-2, as well as Hek293, were measured using the MTT assay. Briefly, approximately 10,000 cancer cells were seeded in a 96-well plate overnight and exposed to increasing concentrations of Pt(TSC)Cl complex and cisplatin (up to 200 µM) for 24, 48, and 72 h. Then, 20 ml of MTT solution (5 mg/ml) was added to every well and kept at 37 °C for 4 h in the dark. After removing the medium, 200 µl DMSO was added, and the solution was kept on a shaker for approximately 15 min until the formazan solved completely. The absorbance was evaluated at 490 nm with an ELISA reader. The IC_50_ was computed by GraphPad Prism 7.0 software.

### Determination of apoptotic cells

Three cancer cell lines were seeded in 6-well plates at 25*10^4^ cells per well and incubated with the IC_50_ values of the Pt(TSC)Cl complex and cisplatin at 48 h. After washing the cells with cold PBS, they were resuspended in 100 µl binding buffer containing Annexin V-FITC (5 μL) and PI (5 μL) solution. After incubation for 15 min at room temperature, cells were washed and suspended in 100 μL of 1 × binding buffer. Cells were investigated by a FACSCanto II (Bio–Rad) flow cytometry system. The percentage of apoptotic cells was calculated by summation of the cells in (annexin V+ , PI−) and (annexin V+ , PI+) and the percentage of necrotic cells achieved from (annexin V−, PI+).

### Investigation of cell cycle progression

Evaluation of cell cycle progression was performed by flow cytometry of propidium iodide and can identify the fraction of time spent by cells in various stages of the cell cycle. First, A549, MCF-7, and Caco-2 cells were seeded in 6-well plates at 25*10^4^ cells per well and exposed to a concentration at the IC_50_ of Pt(TSC)Cl complex and cisplatin for 48 h. Cell cycle distribution was determined by flow cytometry by the protocol described previously^28^. The control condition consisted of cells cultured in the absence of treatment but in the presence of PI. After harvesting the cells with trypsin, they were rinsed and fixed with 70% ethanol and kept for 3 days at 4 °C. Then, the samples were centrifuged, and after removal of supernatants after removing supernatants, the cells were dispersed in 300 µL of PBS. Next, after adding 10 µL of ribonuclease-A (10 mg/mL) and 45 min of incubation, 10 µL propidium iodide (1 mg/mL) was added to each of the samples and vortexed. After 10 min of incubation at room temperature and in the dark, the cells were examined by a FACSCalibur flow cytometer to estimate the cell cycle phases. FlowJo 7.6 software was used to analyze the cell cycle.

### RNA isolation and cDNA synthesis

Three cancer cell lines were seeded in 6-well plates at 10^6^ cells per well and incubated with the IC_50_ values of the Pt(TSC)Cl complex and cisplatin at 48 h. Then, the cells were rinsed with cold PBS and harvested. Total RNA was isolated from MCF-Z, A549, and Caco-2 cell lines by the TRIzol method (TRIzol™ Reagent (Invitrogen, Catalog number: 15596026)). Briefly, the cells were centrifuged at 500 g at 4ºC, the plates were processed for cell lysate by 750 μl of RiboEx; then 200 μl chloroform was subjoined to the cell lysate and after incubating for 2 min at room temperature, they were centrifuged at 12,000 g for 20 min (4 °C). Following collection of aqueous phases, one volume of isopropyl alcohol was dispensed for precipitation of total RNA at 12,000 g for 20 min (4 °C). The RNA plate was rinsed with 75% ethanol and dissolved in deep water. Complementary DNA (cDNA) synthesis was performed by Revert Aid Reverse Transcriptase (Cat numb: EP0441, Thermo Scientific, Lithuania) according to the manufacturer’s instructions.

### Real time PCR

For evaluation of the apoptosis pathway genes, real time PCR was carried out based on the following PCR program^28^: initial denaturation at 95 °C for 15 min, 45 cycles of denaturation at 95 °C for 15 sec, and annealing/extension at 60 °C for 50 sec. The real time PCR mixture contained 5 μL 2x SYBR Green Master Mix (RealQ Plus 2x Master Mix Green, Ampliqon, Denmark), 2 μL cDNA, 0.5 μL of 5 pmol/μL primer pair mix (Eurofin, Germany), and 3 μL H2O. Primers sequences are indexed in Table 1. The glyceraldehyde-3-phosphate dehydrogenase (GAPDH) gene was applied as the reference gene, and the – ∆∆Ct method was utilized to estimate the fold changes.

**Table1:** List of the primers.

Gene	Forward primer (5’-3’)	Reverse primer (5’-3’)
CASP3	GAAATTGTGGAATTGATGC GTGA	CTACAACGATCCCCTCTGA AAAA
CASP-6	ATGGCGAAGGCAATCACAT TT	GTGCTGGTTTCCCCGACAT
CASP-7	AGGGACCGAGCTTGATGAT G	CACTGGGATCTTGTATCGA GGA
CASP-8	GATCAAGCCCCACGATGAC	CCTGTCCATCAGTGCCATA G
CASP-9	CTTCGTTTCTGCGAACTAA CAGG	GCACCACTGGGGTAAGGTT T
CASP-10	AGAAACCTGCTCTACGAAC TGT	GGGAAGCGAGTCTTTCAGA AG
CASP12	TGTTACAAAGGCTCATGTG GAAA	GGGTCAGTATATTTGGGGT CTCA
Bax	TTCTGACGGCAACTTCAAC T	CAGCCCATGATGGTTCTGA T
Bcl-2	GGGAATCGATCTGGAAATC CTC	GGCAACGATCCCATCAATC T
GAPDH	ACAACTTTGGTATCGTGGA AGG	GCCATCACGCCACAGTTTC

### Assessment of apoptotic protein level alterations by western blot analysis

Caco-2 cancer cells, as the most sensitive cells to Pt(TSC)Cl, were seeded in 6-well plates at a population of 10^6^ cells per well and treated with cisplatin and Pt(TSC)Cl for 48 h. Assessment of apoptotic protein level alterations by western blot analysis by the protocol described previously^28^. The control included cells without treatment. To provide the cell lysate, cells were collected and treated with the following materials: RIPA buffer [500 µL of Tris–HCl (pH = 8), one tablet of protease inhibitor cocktail, 0.003 g EDTA, 0.08 g NaCl, 0.025 g sodium deoxycholate, 0.01 g SDS and 10 µl of Triton NP40 (1%)] used at 4 °C. After that, the cells were centrifuged at 12,000 rpm for 10 min at 4 °C. To measure total protein, the supernatant of cells was determined by the Bradford assay (Bio–Rad protein assay, Bio–Rad Laboratories, USA) using a spectrophotometer (Bibby Scientific Ltd, Beacon Rd, UK). The portions of target protein received from the sodium dodecyl sulfate–polyacrylamide gel electrophoresis (SDS–PAGE) were carried over the polyvinylidene difluoride (PVDF) membrane and were blocked with TBST buffer containing 5% (w/v) skim milk (0.1% v/v Tween®20-tris buffered saline: TBST). The blocked PVDF membranes that contained the target proteins were incubated with specific primary antibodies: Bax (B-9) mouse monoclonal antibody (Santa Cruz) (1:1000), Bcl-2 sc-492 (Santa Cruz) rabbit monoclonal antibody (1:1000), caspase-7 (C7) (Cell Signaling) rabbit polyclonal antibody (1:1000), Caspase-3 Cell Signaling rabbit monoclonal antibody (1:1000), caspase-9 (Cell Signaling) rabbit polyclonal antibody (1:1000), GAPDH (Santa Cruz) mouse monoclonal antibody (1:1000) and P27 (Elabscience) rabbit polyclonal antibody (1:400) that were diluted with blocking buffer) overnight at 4 °C. The membrane was washed with TBST and incubated with secondary antibodies (m-IgGκ BP-HRP (Santa Cruz) for Bax (B-9), Bcl-2 and GAPDH, and mouse anti-rabbit IG-HRP (Santa Cruz) was used for Caspase-3, 7, 9 and P27, which were diluted using blocking buffer (1:1000).) for 1 h at room temperature. The target protein bands were visualized by an enhanced chemiluminescence detection kit (Thermo Fisher Scientific, Breda, the Netherlands) and Amersham® Imager 600 system (GE Healthcare Life Sciences, Eindhoven, Netherlands). Finally, the western blotting results were normalized to the expression of GAPDH as the loading control. All the protein bands were quantified by ImageJ software (v. 1.52n).

## Results and discussion

**Scheme 2:**
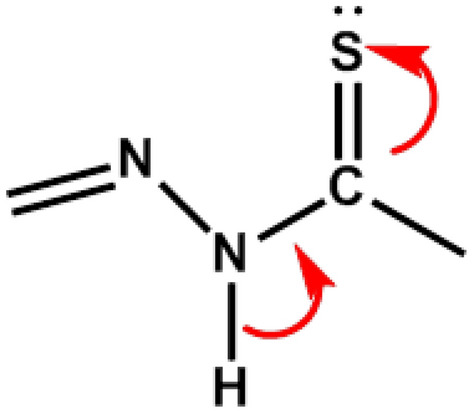
Deprotonation of hydrogen atoms in the complexation process of HTSC.

The reaction of platinum (II) with ligand (HTSC) yielded the corresponding complexes. On the basis of elemental analysis and X-ray structure determination, the complex was formulated as Pt (TSC)Cl. In the complex, the ligand acts as monoanionic through deprotonation of the hydrazonic hydrogen atom (Scheme 2).

### Structural characterization of the Pt(TSC)Cl complex with ^1^HNMR and FTIR

In the ^1^HNMR spectrum of the complex (Fig. S1), the NH-N proton signal at 12.00 ppm in the free ligand was omitted upon coordination with platinum. In addition, the CH=N proton shifted from 8.20 in the free ligand to the downfield region, 8.65 ppm, as a strong singlet in the complexation. In comparison with the IR spectra of the free ligand (HTSC) and Pt(TSC)Cl complex (Fig. S2), the displacement of the bands assigned to ν _C-H_ and ν _N–H_ in the 2927–3440 cm^-1^ region and the bands assigned to ν _C=N_ and ν _C=C_ in the 1440–1700 cm^-1^ region could be indicative of coordination of the ligand through its nitrogen atoms, as the crystal structure shows.

### X-ray crystal structure of Pt(TSC)Cl complex

**Figure 1:**
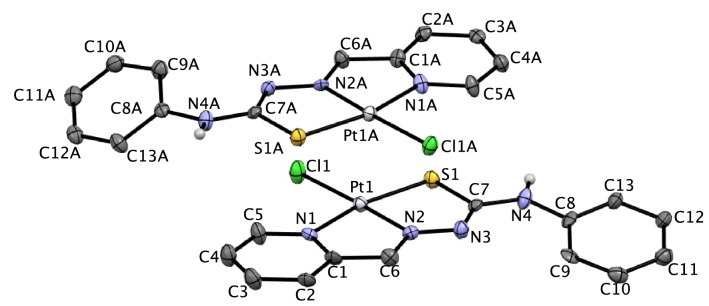
ORTEP diagram of the two independent molecules of platin complex Pt (TSC) Cl; ellipsoids are at the 50% probability level.

Relevant data for the collection and structure solutions are listed in Table 2, and Fig. 1 shows the thermal ellipsoid plot of the complex molecules with an atom numbering scheme. The Pt(TSC)Cl complex crystallizes in the orthorhombic space group *Pna2*_*1*_ with two independent molecules in the asymmetric unit, which form a π-stacked dimer where the distance between each of the planes is defined by atoms C1, C7, N1, N3, and Pt1, and the Pt atom of the second molecule is 3.521(4) Å. Selected bond distances and bond angles are summarized in Tables 3 and 4, respectively. Small differences in the geometrical parameters for the two molecules presumably arise due to differences in their packing environments in the crystal. These two molecules are essentially identical, and herein after, the values for molecule 1 and molecule 2 are presented in brackets. The most significant difference is in the torsional angle C7–N4–C8–C9 (7(3)[− 31(3)])°, which shows that the complex is not a rigid molecule and that the phenyl ring can twist around the C7–N4 bond, which may affect the biological behavior of the complex. There are four Pt(II) centers in the complex molecules, coordinated by one chlorine ion and by three donor atoms of the organic ligand (N-pyridine, N-imine and S). The ligand acts as a monoanionic ligand (TSC) through deprotonation of the hydrazonic hydrogen atom of the free ligand upon complexation (Scheme 2).

**Table 2:** Crystallographic data and structure refinement parameters for the complex [Pt(TSC)Cl].

Chemical formula	C_13_H_11_ClN_4_PtS
Formula weight	485.86
Crystal system	Orthorombic
Space group	Pna2_1_
a/Å	25.9209(4)
b/Å	7.5033(1)
c/Å	14.0772(2)
V/Å	2737.91(7)
Z	8
T/K	100
Density (calc.) (g/cm)	1.5899
Radiation Type	CuKα (λ = 1.54184 Å)
µ/mm-1	22.35
Crystal size (mm)	0.14 × 0.02 × 0.02
T_min_, T_max_	0.470, 1.000
Number of measured reflections	10,357
Number of independent reflections	4650
Number of reflections I > 2σ (I)	4461
θ max	77.1°
θ min	4.6°
Index ranges/h/k/l	− 27 < h < 32, − 5 < k < 9, − 17 < l < 17
(sin θ/λ)_max_ (Å^-1^)	0.632
F(ooo)	1824
Rint	0.091
R[F^2^ > 2σ(F^2^)]	0.052
WR(F^2^)]	0.157
(∆/σ)_max_	0.001
∆P_max_(eA^-3^)	3.13
∆P_min_(eA^-3^) CCDC number	− 2.96 2,109,926
W = 1/[σ^2^(F_0_^2^) + (0.1179P)^2^ + 21.1873P	Where *P* = (F_0_^2^ + 2F_c_^2^)/3

**Table 3:** Selected bond length (Å) Molecule 2 corresponds to the molecule labeled with prefix ‘A’ in Fig. 1.

Bond lengths	Molecule 1	Molecule 2
Pt_1_–S_1_	2.264 (5)	2.255 (5)
Pt_1_–Cl_1_	2.314 (5)	2.322 (4)
Pt_1_–N_1_	2.055 (15)	2.048 (16)
Pt_1_–N_2_	1.986 (16)	1.953 (16)
S_1_–C_7_	1.800 (19)	1.775 (18)
C_7_–N_3_	1.28 (3)	1.33 (2)
N_3_–N_2_	1.32 (2)	1.38 (2)
N_2_–C_6_	1.31 (3)	1.34 (3)
C_6_–C_1_	1.46 (3)	1.46 (3)
C_1_–N_1_	1.36 (3)	1.36 (3)
N_4_–C_8_	1.42 (2)	1.44 (2)

**Table 4:** Selected bond angles (°).

	Molecule 1	Molecule 2
N_1_–Pt_1_–N_2_	81.3 (6)	80.8 (6)
N_1_–Pt_1_–Cl_1_	95.0 (4)	96.7 (5)
N_1_–Pt_1_–S_1_	164.9 (4)	165.1 (5)
N_2_–Pt_1_–Cl_1_	176.2 (5)	177.5 (5)
N_2_–Pt_1_–S_1_	83.6 (5)	84.3 (5)
S_1_– Pt_1_–Cl_1_	100.14 (18)	98.17 (18)
Pt_1_–S_1_–C_7_	94.4 (6)	95.1 (6)
S_1_–C_7_–N_3_	123.6 (15)	124.9 (14)
C_7_–N_3_–N_2_	114.5 (17)	110.3 (15)
N_3_–N_2_–C_6_	122.0 (16)	118.4 (16)
Pt_1_–N_2_–C_6_	114.2 (13)	116.3 (13)
N_2_–C_6_–C_1_	118.1 (18)	114.7 (17)
C_6_–C_1_–N_1_	113.7 (17)	115.4 (17)
C_1_–N_1_–Pt_1_	112.6 (13)	112.6 (12)
S_1_–C_7_–N_4_	111.4 (14)	113.7 (14)
N_4_–C_7_–N_3_	124.8 (18)	121.3 (17)
C_7_–N_4_–C_8_	127.6 (17)	127.5 (17)

The Pt atoms in each molecule have a slightly distorted square-planar coordination geometry with τ_4_ indexing of 0.12[0.13] (τ_4_ = [360 (α + β)]/141, where α and β are the two largest coordination angles, τ_4_ values are zero and unity for perfect square-planar and tetrahedral geometries, respectively^29^. The length of C7–S bonds in these complex molecules which are 1.800 (19)Å [1.775(18)Å] The length of C7–S bonds in these complex molecules, which are 1.800 (19) Å [1.775(18) Å], shows an increase in comparison to the C-S bond in the free ligand[1.677(2) Å]^30^ and is indicative of substantial single-bond characters.

**Figure 2:**
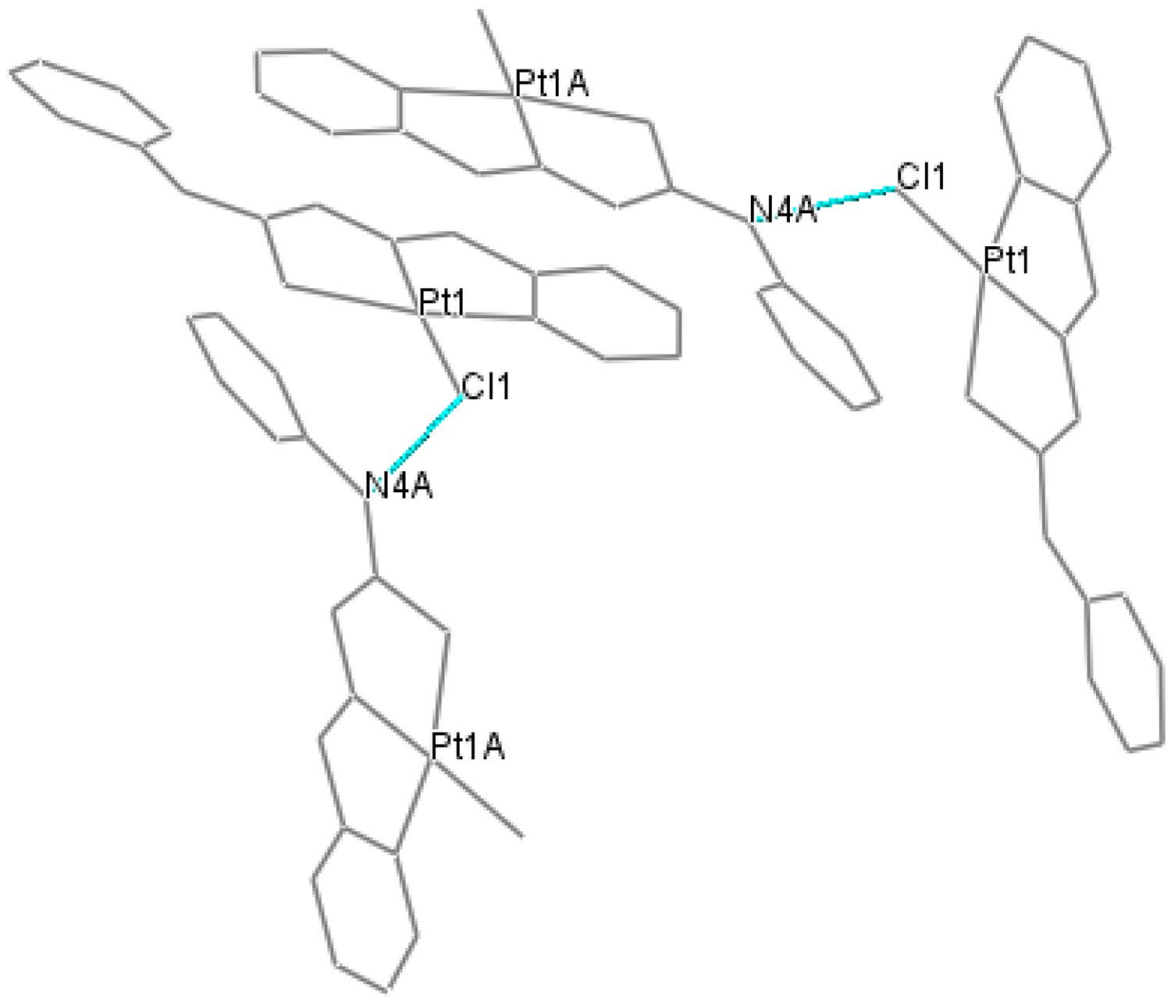
Hydrogen bonding between adjacent molecules of neighboring asymmetric unit cells.

The C7–N3 bond lengths, (1.28(3) [1.33(2)]Å, are decreased compared to the corresponding distance in the free ligand (1.3685(2) Å^3^). The shortening of the mentioned C–N bonds and lengthening of the C–S bonds in the complex molecules compared to the corresponding bonds in the free ligand revealed that complexation occurs by losing the hydrogen atom attached to N3 and N3A, as shown in Fig. 1. The shortening of C7–N3 is more than the shortening of C7A–N3A, and vice versa, the length of S1–C7 is more than the lengths of S1A–C7A. Therefore, the double and single characters of C7–C3 and S1–C7 bonds, respectively, are more than those of C7A–C3A and S1A–C7A bonds, so the N3 atom has a more negative charge than the N3A atom. There is no interaction between the two molecules in one asymmetric unit cell, but each molecule is in insignificant short contact with adjacent molecules in the neighboring asymmetric unit cells. The Cl1 atom in the molecule is linked with N4A of an adjacent molecule in the neighboring asymmetric unit cell by hydrogen bonding (Fig. 2), Cl1……H4AN4A with a distance of Cl…. N = 3.510 Å, Cl……. H = 2.715 Å and with angle ClHN = 154.36°.

### Cell viability assay in vitro

To investigate the potency of the Pt(TSC)Cl complex as an anticancer drug, three different cancer cell lines, MFC-7, A549 and Caco-2 (10,000 cells per well), were exposed to increasing concentrations of Pt(TSC)Cl and cisplatin (up to 200 µM) to determine their IC_50_ values/µM (Fig. S3 and Table 5). All of cell lines were treated for three different duration times (24, 48 and 72 h). The Caco-2 cells were very sensitive to Pt(TSC)Cl in comparison with the two others in all three different durations of treatment. We also used cisplatin, a commercially available platinum-based compound, as a reference. In the Caco-2 cell line, the Pt(TSC)Cl complex was highly toxic, with an IC_50_ < 10 µM, and even the toxicity was higher than that of cisplatin in all durations of the cell viability assay (****, *p* < 0.00001). After two days of treatment for 48 h, the Pt(TSC)Cl complex showed 46-fold more toxicity than cisplatin in the Caco-2 cell line. In the MCF-7 and A549 cell lines, cisplatin showed more toxicity than the Pt(TSC)Cl complex (***, *P* ≤ 0.001). Cisplatin’s lower toxicity to Caco-2 cell lines compared to MCF7 and A549 cell lines was confirmed in our previous work^31^.

**Table 5:** The IC_50_ value of Pt(TSC)Cl complex and cisplatin on cancer and normal cell lines.

IC_50_ (µM)									
Cell lines	24 h			48 h			72 h		
	Cis	Pt(TSC)Cl complex	*P* value	Cis	Pt(TSC)Cl complex	*P* value	Cis	Pt(TSC)CI complex	*P* value
A549	96.8	146.6		42.93	101.7		13.12	69.28	
MCF-7	162	200		142	193		150	181	
Caco-2	149	17.2	< 0.00001	107	2.3	< 0.00001	37.32	1.75	< 0.00001
Hek293	4.15	63.9	< 0.00001						

*Cis: Cisplatin, A549: Human lung cancer cells, Caco-2: Human colon cancer cells, MCF-7 Human breast cancer cells and Hek293: Human embryonic kidney cells.

Since renal toxicity is a major problem with platinum-based drugs, the toxic effect of the Pt(TSC)Cl complex was evaluated on Hek293 kidney cells in comparison to cisplatin. The Pt(TSC)Cl complex, with the higher cytotoxic potency for the Caco-2 cell line, revealed lower nephrotoxicity in Hek293 cells (***p < 0.0001) (Fig. S3) than cisplatin (cisplatin was 15-fold more toxic to Hek293 cells than the Pt(TSC)Cl complex). A summary of all cytotoxicity data is listed in Table 5.

Three different cancer cell lines, MCF-7, A549, Caco-2, and normal Hek293 cells, were treated with different concentrations of cisplatin and Pt(TSC)Cl complex for 24, 48, 72 h. Cell viability was assessed with MTT and is presented relative to untreated cells (set to 100%).

### Arresting the cell cycle in sub G1, another proof of programmable cell death in the presence of the Pt(TSC)Cl complex

**Figure 3:**
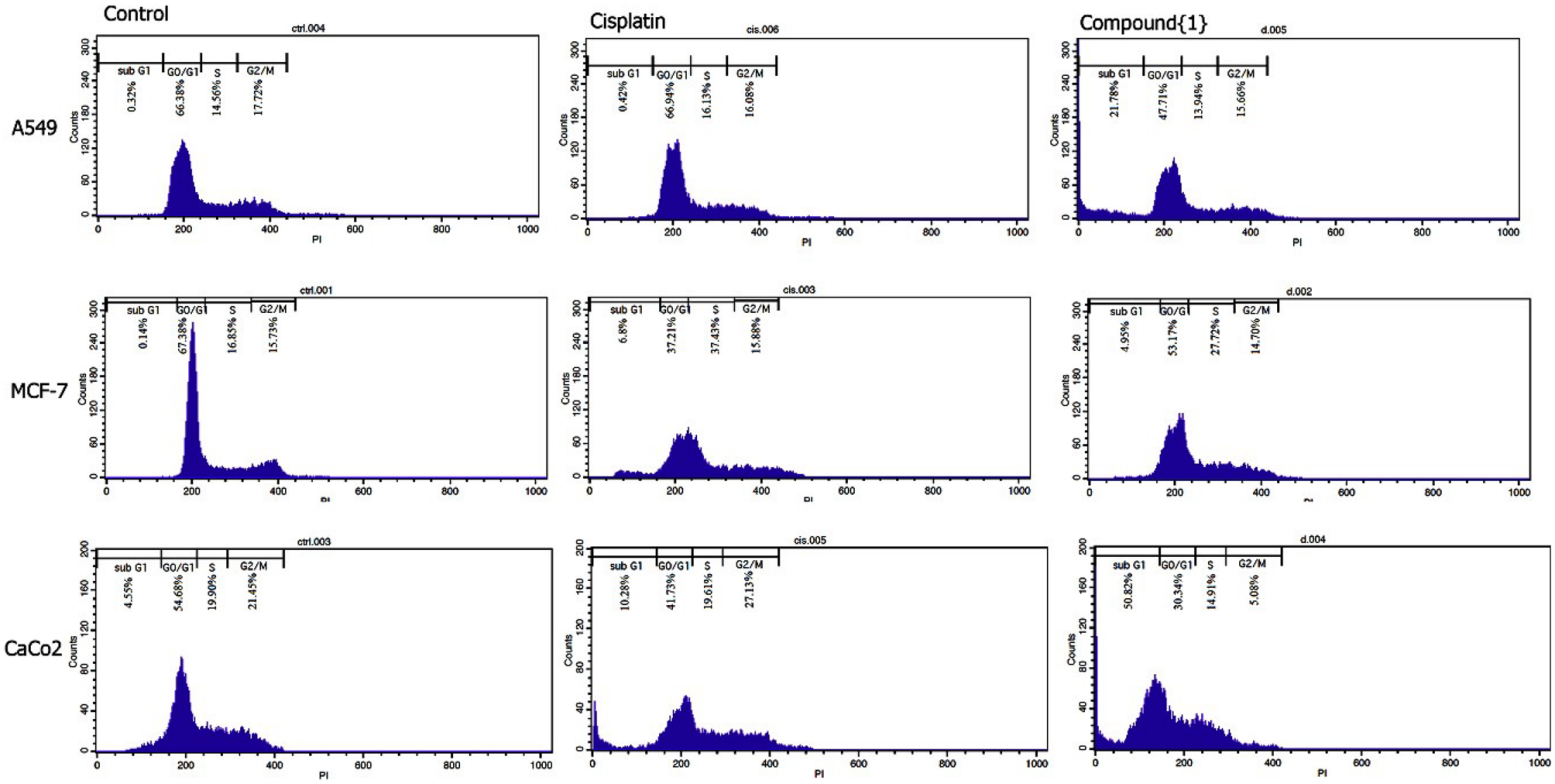
Cell cycle distribution of MCF-7, A549 and Caco-2 cells treated with the indicated IC_50_ concentrations of Pt(TSC)Cl complex (compound{1}) and cisplatin for 48 h determined by staining the DNA content of the cell followed by flow cytometry. The percentage of cells in G0/G1, S or G2/M phase is indicated.

As shown in Fig. 3 and Table 6, untreated A549, MCF-7 and Caco-2 cells in the G0/G1, S or G2/M phases showed normal growth conditions. In the MCF-7 cells treated with cisplatin (S: 37.2%) and Pt(TSC)Cl complex (S: 28%), the amount of S phase significantly increased compared with control cells (*p* < 0.0001). Therefore, S phase arrest was dominant in MCF-7 cells treated with both cisplatin and the Pt(TSC)Cl complex. Emerging sub G1 phase as a marker for apoptotic cells occurred in the Caco-2 cell lines treated with cisplatin and Pt(TSC)Cl complex. Compared to the nontreated Caco-2 cell lines (control group), in groups that were treated with cisplatin and Pt(TSC)Cl, approximately 10 and 51% of the cells were observed in the subG1 phase, respectively. The difference between subG1 arrest in the presence of the Pt(TSC)Cl complex and cisplatin and between the Pt(TSC)Cl complex and control cells was significant (*p* < 0.0001). These data are also in line with the results of Annexin V and P analysis. However, in A549 cells, just in the presence of Pt(TSC)Cl complex was the sub G1 phase observed (subG1:22%). The increase in cell number at the subG1 phase can be associated with apoptotic cell death, leading to reduced cell entry to the G2/M and S phases^32^. Observing approximately 50% cell arrest of Caco-2 cells also proved that in the presence of the Pt(TSC)Cl complex, apoptosis was a dominant pathway of cell death (Fig. 3 and Table 6). In support of this claim, we noted that the percentage of proliferating cells at the G2/M phase was diminished in groups that were treated with Pt(TSC)Cl complex.

**Table 6:** Quantitative results of Cell cycle distribution of MCF-7, A549 and Caco-2 cells treated with the indicated IC_50_ concentrations of cisplatin and Pt(TSC)Cl complex for 48 h determined by staining the DNA content of the cell followed by flow cytometry. The percentage of cells in G0/G1, S or G2/M phase is indicated.

	A549			MCF-7			Caco-2		
	Control	Cis	Pt(TSC)Cl complex	Control	Cis	Pt(TSC)Cl complex	Control	Cis	Pt(TSC)Cl complex
SubG1	0.32	0.42	21.78	0.14	6.8	4.95	4.55	10.28	50.82
G0/G1	66.38	66.94	47.71	67.38	37.21	53.17	54.68	41.73	30.34
S	14.56	16.13	13.94	16.85	37.43	27.72	19.90	19.61	14.91
G2/M	17.72	16.08	15.66	15.73	15.88	14.70	21.45	27.13	5.08

*Cis: Cisplatin, A549: Human lung cancer cells, Caco-2: Human colon cancer cells, MCF-7 Human breast cancer cells.

### Apoptosis induction assessed by flow cytometry of Annexin V, PI

**Figure 4:**
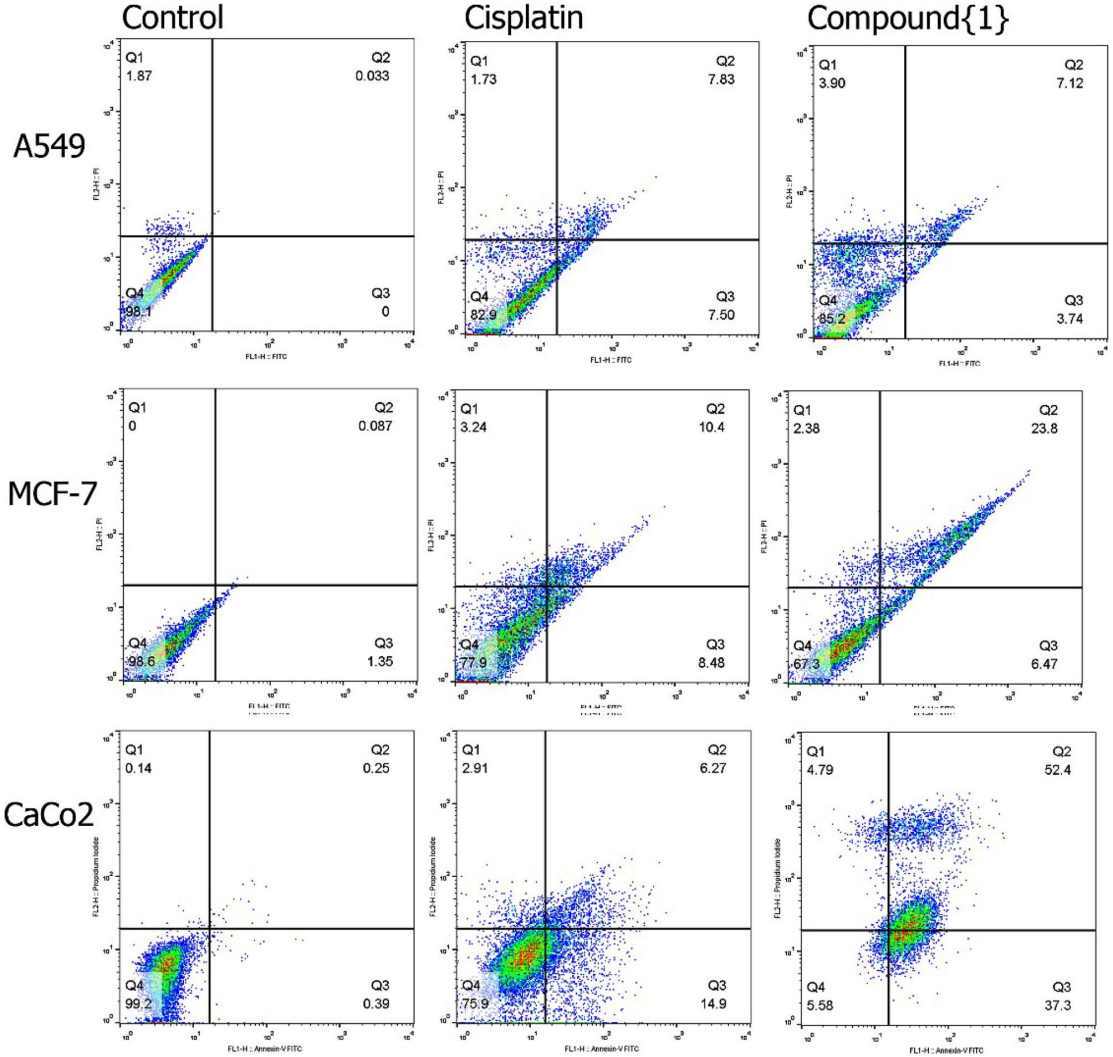
The apoptotic effects of treatments composed of cisplatin and Pt(TSC)Cl complex in A549, MCF-7 and Caco-2 cells were determined by flow cytometry. Untreated cells were considered as control.

An Annexin-V/PI assay was performed via flow cytometry to measure the percentage of cells with early and late apoptotic changes. The percentages of apoptotic and necrotic cells for three cancer cell lines in the presence of both Pt(TSC)Cl complex and cisplatin were compared with those for vehicle control (stained viable cells) and are reported in Fig. 4 and Table 7. To gain insight into the chosen pathway in the presence of Pt(TSC)Cl complex and cisplatin, the percentage of necrotic cells was compared to the percentage of apoptotic cells. The results showed that Caco-2 cells treated with Pt(TSC)Cl complex were mostly killed by the apoptosis pathway provided by 89.7% apoptotic vs 4.79% necrotic cells after 48 h. It seems that apoptosis is the hallmark of Caco-2 cell lines death, in which approximately 37 and 52% of cells showed early and late apoptosis, respectively. Consistent with our data, late apoptotic changes were more evident cytotoxic effects in cells that were treated with Pt(TSC)Cl complex. In the presence of cisplatin, 21.17 and 2.91% of cells were killed by apoptosis and necrosis, respectively. In the A459 cells exposed to cisplatin and Pt(TSC)Cl complex, the percentages of apoptotic and necrotic cells were 15.33 and 1.73 and 10.86 and 3.90, respectively, which confirmed that they did not show significant toxicity to A549 cell lines. In MCF-7 cell lines treated with Pt(TSC)Cl complex, 31.28 and 2.37% of cells were killed by apoptosis and necrosis, respectively, and this percentage decreased to 18.88 and 3.24 when MCF-7 cells were treated with cisplatin. The results showed that in Caco-2 cell lines, the most common pathway in the presence of the Pt(TSC)Cl complex is apoptosis, so it can be shown that the Pt(TSC)Cl complex has one or more targets in Caco-2 cancer cells, and by targeting them, programmable cell death and apoptosis can be modulated.

**Table 7:** Quantitative results of apoptotic effects of A549, MCF7 and Caco-2 cells, determined by Annexin V/FITC assay, for cells treated with IC_50_ concentration of Cisplatin and Pt(TSC)Cl complex. Cells with no treatment considered as control group.

	A549			MCF-7			Caco-2		
	Control	Cis	Pt(TSC)Cl complex	Control	Cis	Pt(TSC)Cl complex	Control	Cis	Pt(TSC)Cl complex
Viable (PI^-^/FITC^-^)	98.1	82.9	85.2	98.6	77.9	67.3	99.2	75.9	5.58
Early apoptosis (PI^-^/FITC^+^)	0	7.50	3.74	1.35	8.48	6.47	0.39	14.9	37.3
Late apoptosis (PI^+^/FITC^+^)	0.033	7.83	7.12	0.087	10.4	23.8	0.25	6.27	52.4
Necrosis (PI^+^/FITC^-^)	1.87	1.73	3.9	0	3.24	2.38	0.14	2.91	4.79

*Cis: Cisplatin, A549: Human lung cancer cells, Caco-2: Human colon cancer cells, MCF-7 Human breast cancer cells.

### Apoptosis induction assessed by real time PCR

**Figure 5:**
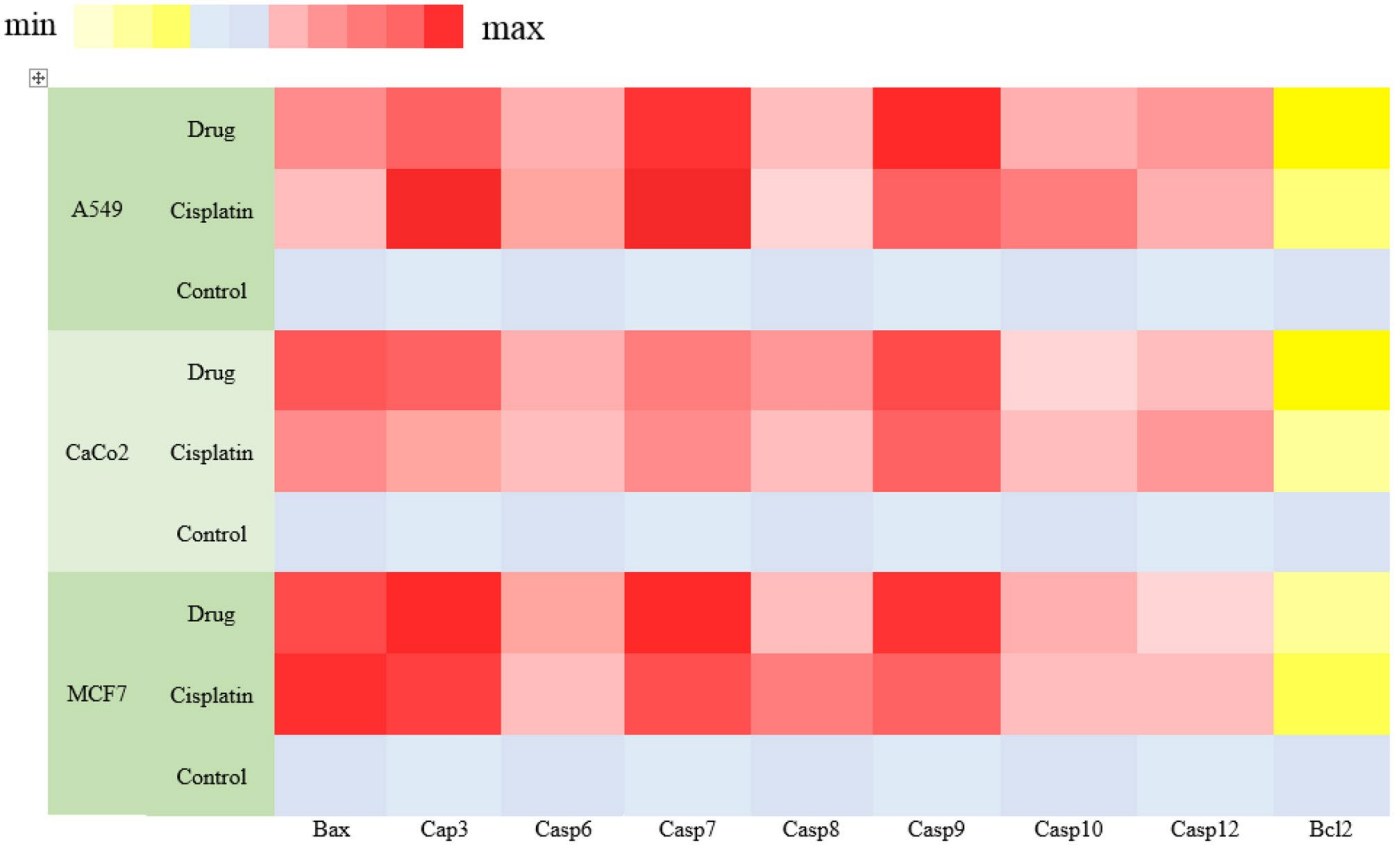
Real time PCR expression of apoptosis panel. The heatmap indicates the range of expression of apoptosis pathway-mediated genes in different cells and treatments. Bcl2 was down regulated in almost all samples; however, the expression of Bax, Casp3, Casp7, and Casp9 was upregulated, which suggests activation of the intrinsic apoptosis pathway. Drug: Pt(TSC)Cl complex, Control: untreated cells.

With the aim of precise investigation of the apoptosis pathway, real time PCR was performed. Bax, Bcl2, and different caspase subsets participate in regulating the apoptotic response. To this end, we measured the gene expression of different factors using real time PCR analysis. The results of the real-time PCR test are presented as a heatmap of the change in gene expression related to the control group (Fig. 5). Upregulation of Caspase 9 is related to mitochondrial injury and can trigger an internal apoptosis signaling pathway. In the next step, Caspase 9 activates the common apoptosis pathway consisting of Caspases 3 and 7. The data showed that the expression of apoptotic pathway genes, such as Bax and Caspases 9, 3, and 7, was upregulated in the cell treatment group that received Pt(TSC)Cl complex and cisplatin compared to the control. Low level of Bcl-2 mRNA is in agreement with apoptosis. Additionally, apoptotic gene expression was more dominant in all three cell lines treated with the Pt(TSC)Cl complex compared to cisplatin, which is in agreement with the cell cycle and Annexin-V analysis. The real time PCR results for apoptosis pathway genes showed that the expression pattern benefits from the intrinsic apoptosis pathway through the Bax/Bcl-2-Casp9-Casp3/Casp7 axis.

### Western blotting to evaluate the mitochondrial mediator apoptotic pathway

**Figure 6:**
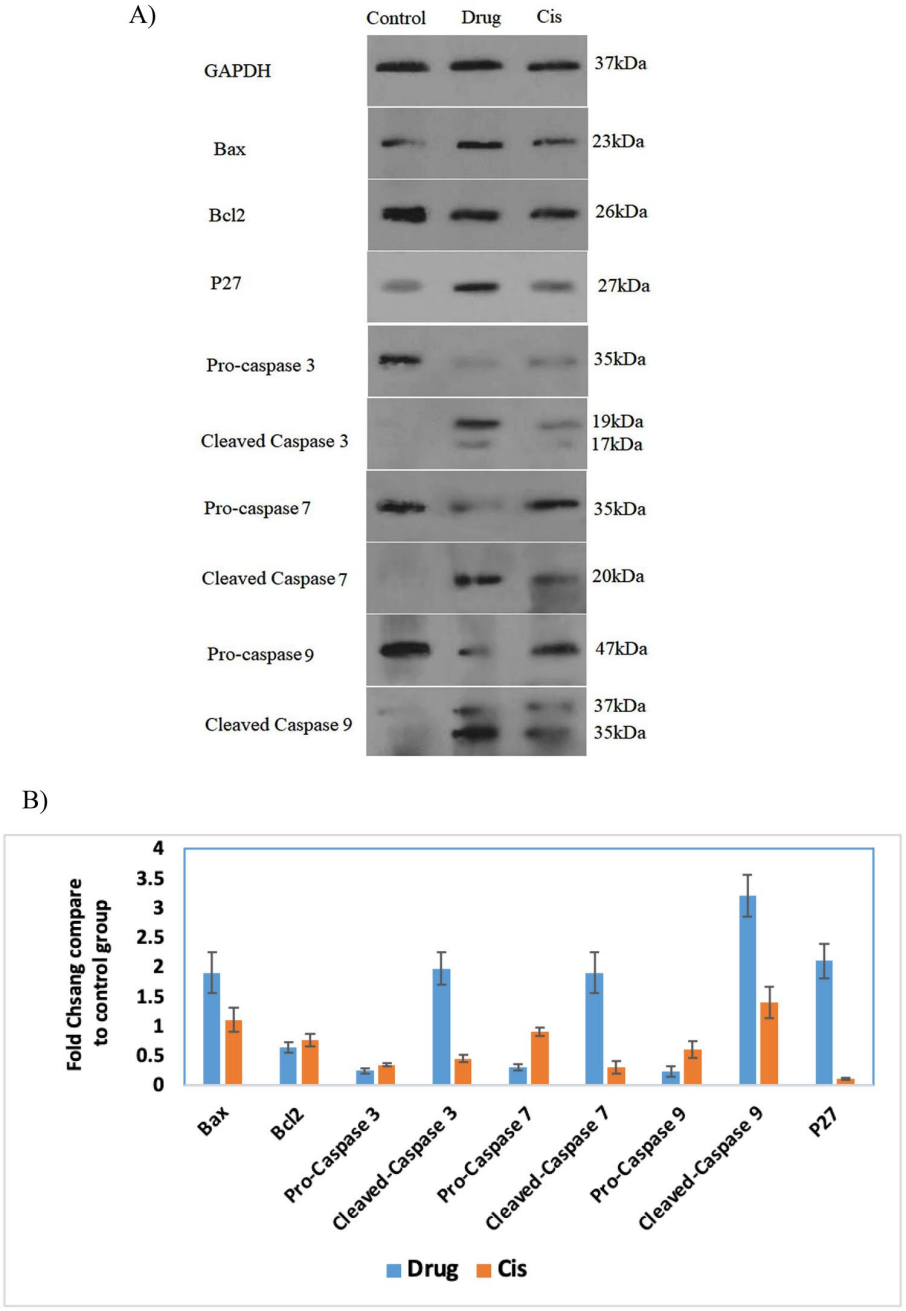
The cropped blots/gels of proteins expression obtained from western blotting of the Caco-2 cells treated with IC_50_ value of Cisplatin and Pt(TSC)Cl complex. Un-treated cells were considered as the control group. Proteins: Bcl-2, Bax, p27, pro-Caspase-3, Cleaved-Caspase-3, pro-Caspase-7, Cleaved-Caspase-7, pro-Caspase-9, Cleaved-Caspase-9, and GAPDH as internal control. Full-length blots/gels are presented in Supplementary Fig. S4 B) Quantitative results of apoptotic effects assessed by western blot analysis. Drug: Pt(TSC)Cl complex, Cis: Cisplatin.

Some studies have shown that mitochondrial DNA damage is evident in cisplatin-treated cells^31^. To check the activation of the mitochondria-dependent apoptosis pathway, the expression of BAX, a proapoptotic protein, and BcL-2, an antiapoptotic protein, was evaluated by western blotting. Overexpression of the BAX gene at the protein level in the presence of cisplatin and the Pt(TSC)Cl complex was observed. This protein can oligomerize and permeabilize the mitochondrial outer membrane to some apoptogenic factors. However, antiapoptotic proteins such as Bcl-2 can prevent the release of mitochondrial apoptogenic factors. The higher amount of Bcl-2 protein in the presence of the Pt(TSC)Cl complex shows that this compound, like cisplatin, can stimulate apoptosis by regulating the antiapoptotic and proapoptotic Bcl-2 protein families. Western blot analysis was performed to assess the executive apoptotic protein levels. Western blot analysis proved that the apoptotic process occurred via cascade activation of Bax, caspase 9, caspase 3 and caspase 7 (Figs. 6 and S4). The protein levels of Bax, caspase 9, caspase 3, and caspase 7 were upregulated and enlarged 1.9-, 3.2-, 1.97- and 1.92-fold, respectively, in Caco-2 cells treated with Pt(TSC)Cl complex compared to the control group. Furthermore, the abovementioned procedure was proven by the downregulation of Bcl2 protein levels. This result was in agreement with real time PCR outcomes. As reported previously, p27 protein has two duties in the determination of cell futures: pro-apoptotic function and cell cycle arrest^33^. According to our results, a 2.14-fold increase in p27 protein in Caco-2 cells treated with Pt(TSC)Cl complex compared to the control group showed the occurrence of apoptosis in Caco-2 cell lines treated with Pt(TSC)Cl complex. The fold changes of the mentioned proteins in Caco-2 cells treated with cisplatin were not increased significantly compared to untreated Caco-2 cells. The western blot results were in accordance with the cell cycle, Annexin-V and real time PCR results, which showed the highest apoptotic performance of CaCo-2 cells treated with Pt(TSC)Cl complex compared to cisplatin.

Despite treating CRC with different kinds of therapies, it remains one of the most aggressive cancers, with a poor prognosis and high mortality. Platinum drugs, mostly cisplatin, have been conventionally used in chemotherapy against CRC. To fight against cancer, attempts have to be focused on the design and fabrication of very effective antitumor compounds with less toxicity to normal cells and tissues^10,34^. However, because of the substantial adverse effects of platinum-based drugs, as well as tumor resistance to treatment, new thiosemicarbazone derivatives in complex with platin (Pt(TSC)Cl complex) have been designed, and the data gathered and displayed in this work indicate that the Pt(TSC)Cl complex could be a promising substitute in the future. The Pt(TSC)Cl complex displayed potent antiproliferative activity against MCF-7, A549 and Caco-2 cell lines. Previous studies showed that platinum metal complexes of 2-acetylpyridine thiosemicarbazones showed slight antitumor potency against Ehrlich ascites tumor cells in vitro (because of the low solubility of the complex) but potent cytotoxic activity in vivo^35^. Thiosemicarbazone derivative (2-acetylpyridine thiosemicarbazones and heterocyclic thiosemicarbazones)-based complexes with transition metal (palladium (II), platinum (II), ruthenium (III), rhodium (III) and iridium (III) copper (II)) appeared to be more toxic in vivo^35^. This had also been observed earlier for complexes with similar ligands^36^. The representative Pt(TSC)Cl complex exhibited highly potent antiproliferative activity toward the Caco-2 cancer cell lines and lower nephrotoxicity against normal kidney cells, so a higher selectivity for a tumor mass than noncancerous cells was displayed. In agreement with our results, pyridine-2-carbaldehyde thiosemicarbazone and pyridine-2-carbaldehyde 4 N-methylthiosemicarbazone and their complexes with different transition metal ions have antitumor activity in colon cancer cells (HT-29 and SW-480)^37^. Therefore, these kinds of complexes could be replaced with cisplatin since nephrotoxicity is a serious and dose-limiting toxicity of cisplatin^5^. Our results confirmed that the Pt(TSC)Cl complex induced apoptosis by sub-G1 arrest, leading to blockade of cell cycle progression.

Chemotherapeutic agents are mostly used to settle cellular hemostasis by damaging nuclear DNA and inducing cell death by apoptosis. A number of studies checked the DNA interactions of platinum-based compounds, but few of them checked the effect of these compounds on the activation of mitochondrial mediator apoptotic pathways^31^. Apoptotic gene expression was more dominant in all three cell lines treated with the Pt(TSC)Cl complex compared to cisplatin, which is in agreement with the cell cycle and Annexin-V analysis. The real time PCR results for apoptosis pathway genes revealed that the expression pattern benefits from the intrinsic apoptosis pathway via the Bax/Bcl-2-Casp9-Casp3/Casp7 axis. Moreover, more Bcl-2 protein in the presence of the Pt(TSC)Cl complex showed that this compound, like cisplatin, can stimulate apoptosis by regulating the antiapoptotic and proapoptotic Bcl-2 protein families. According to our results, a 2.14-fold increase in p27 protein in Caco-2 cells treated with Pt(TSC)Cl complex compared to the control group showed the occurrence of apoptosis in Caco-2 cell lines treated with Pt(TSC)Cl complex. The western blot results were in accordance with the cell cycle, Annexin-V and real time PCR results, which showed the higher apoptotic performance of Caco-2 cells treated with Pt(TSC)Cl complex compared to cisplatin. Collectively, the Pt(TSC)Cl complex showed selective toxicity against Caco-2 cancer cells, blocking cell cycle progression and inducing apoptosis via the intrinsic pathway. Our findings suggested that the Pt(TSC)Cl complex with the structure of thiosemicarbazone in complex with platinum may be a suitable anticancer agent for colon cancers for further optimization and development.

## Conclusion

In the present study, a new Pt(II) metal complex [Pt(TSC)Cl] of N(4)-phenyl-2-formylpyridine thiosemicarbazone (HTSC) was synthesized and characterized. The X-ray crystal structure shows deprotonation of the ligand upon complexation. The coordination geometry around the four-coordinated Pt atoms in the complex molecule with one Cl atom, two N atoms and one S atom is a slightly distorted square-planar geometry. We found that this complex exerts increased cytotoxicity in Caco-2 cell lines and has reduced nephrotoxic potential compared to cisplatin. The new Pt(TSC)Cl complex was toxic to cancer cell lines in a cell cycle-dependent manner and induced cell death via caspase-mediated apoptosis, as proven at the gene and protein levels. Last, the Pt complex designed in this study may be motivating nominees for the improvement of a new category of anticancer drugs, especially for colorectal cancers. Forthcoming studies should focus on investigating their effectiveness on cisplatin-resistant tumors and their specificity, safety and pharmacokinetic behaviors in vivo.

## Supplementary Information

Supplementary Information.

## References

[1] Rahimi E (2020). Evaluation of demographic, pathologic, and clinical characteristics and overall survival of patients with colon cancer in Northern Iran (Mazandaran Province) during 2012–2019. Gastroenterol. Hepatol. Bed Bench.

[2] Farhood B (2019). A review of incidence and mortality of colorectal, lung, liver, thyroid, and bladder cancers in Iran and compared to other countries. Contemp. Oncol..

[3] Shadmani, F.K., et al., *Geographic distribution of the incidence of colorectal cancer in Iran: a population-based study.* Epidemiology and health, 2017. **39**. 10.4178/epih.e2017020 PMC5543296 28774167

[4] Hartmann JT (2000). A randomized trial comparing the nephrotoxicity of cisplatin/ifosfamide-based combination chemotherapy with or without amifostine in patients with solid tumors. Invest. New Drugs.

[5] Miller RP (2010). Mechanisms of cisplatin nephrotoxicity. Toxins.

[6] Sastry J, Kellie SJ (2005). Severe neurotoxicity, ototoxicity and nephrotoxicity following high-dose cisplatin and amifostine. Pediatr. Hematol. Oncol..

[7] Mármol I (2019). Gold as a possible alternative to platinum-based chemotherapy for colon cancer treatment. Cancers.

[8] Ay B (2020). Antitumor effects of novel nickel–hydrazone complexes in lung cancer cells. New J. Chem..

[9] Neidle, S. *Cancer Drug Design and Discovery* (Elsevier, 2011).

[10] He Z (2019). Novel thiosemicarbazone derivatives containing indole fragment as potent and selective anticancer agent. Eur. J. Med. Chem..

[11] Pape VFS (2016). Design, synthesis and biological evaluation of thiosemicarbazones, hydrazinobenzothiazoles and arylhydrazones as anticancer agents with a potential to overcome multidrug resistance. Eur. J. Med. Chem..

[12] Heffeter P (2019). Anticancer thiosemicarbazones: chemical properties, interaction with iron metabolism, and resistance development. Antioxid. Redox Signal..

[13] Nishida CR, Ortiz de Montellano PR (2011). Bioactivation of antituberculosis thioamide and thiourea prodrugs by bacterial and mammalian flavin monooxygenases. Chem. Biol. Interactions.

[14] Šarkanj B (2013). 4-Methyl-7-hydroxycoumarin antifungal and antioxidant activity enhancement by substitution with thiosemicarbazide and thiazolidinone moieties. Food Chem..

[15] Summers KL (2019). A structural chemistry perspective on the antimalarial properties of thiosemicarbazone metal complexes. Mini. Rev. Med. Chem..

[16] Elsayed HE (2017). Rationally designed hecogenin thiosemicarbazone analogs as novel MEK inhibitors for the control of breast malignancies. Bioorg. Med. Chem..

[17] Yu Y (2006). Chelators at the cancer coalface: desferrioxamine to Triapine and beyond. Clin. Cancer Res..

[18] Mrozek-Wilczkiewicz A (2019). Anticancer activity of the thiosemicarbazones that are based on di-2-pyridine ketone and quinoline moiety. Eur. J. Med. Chem..

[19] Parker EN (2015). Synthesis and biochemical evaluation of benzoylbenzophenone thiosemicarbazone analogues as potent and selective inhibitors of cathepsin L. Bioorg. Med. Chem..

[20] Zeglis BM, Divilov V, Lewis JS (2011). Role of metalation in the topoisomerase IIα inhibition and antiproliferation activity of a series of α-heterocyclic-N4-substituted thiosemicarbazones and their Cu (II) complexes. J. Med. Chem..

[21] Hu B (2017). Thiosemicarbazone-based selective proliferation inactivators inhibit gastric cancer cell growth, invasion, and migration. MedChemComm.

[22] Serda M (2014). Exploring the anti-cancer activity of novel thiosemicarbazones generated through the combination of retro-fragments: Dissection of critical structure-activity relationships. PLoS ONE.

[23] Kaur H, Gupta M (2018). *Recent advances in thiosemicarbazones as anticancer agents. IJPCBS.

[24] Sheldrick G (2015). Crystal structure refinement with SHELXL. Acta Crystallogr. Sect. C.

[25] Macrae CF, Bruno IJ, Chisholm JA, Edgington PR, McCabe P, Pidcock E, Rodriguez-Monge L, Taylor R, van de Streek J, Wood PA (2008). Mercury CSD 2.0–new features for the visualization and investigation of crystal structures. J. Appl. Cryst..

[26] Farrugia LJ (1999). WinGX suite for small-molecule single-crystal crystallography. J. Appl. Cryst..

[27] Amendola V (2008). Metal-controlled anion-binding tendencies of the thiourea unit of thiosemicarbazones. Chem. Eur. J..

[28] Rahmani A, Rahimi F, Iranshahi M, Kahroba H, Zarebkohan A, Talebi M, Salehi R, Zavvar Mousav H (2021). Co-delivery of doxorubicin and conferone by novel pH-responsive β-cyclodextrin grafted micelles triggers apoptosis of metastatic human breast cancer cells. Sci. Rep..

[29] Yang L, Powell DR, Houser RR (2007). Structural variation in copper(i) complexes with pyridylmethylamide ligands: structural analysis with a new four-coordinate geometry index, τ4†. Dalton Trans..

[30] Lessa JA (2011). Antimony(III) complexes with pyridine-derived thiosemicarbazones: Structural studies and investigation on the antitrypanosomal activity. Polyhedron.

[31] Abyar S (2019). In vitro nephrotoxicity and anticancer potency of newly synthesized cadmium complexes. Sci. Rep..

[32] Rahimi M (2017). Dendritic chitosan as a magnetic and biocompatible nanocarrier for the simultaneous delivery of doxorubicin and methotrexate to MCF-7 cell line. New J. Chem..

[33] Katayose Y (1997). Promoting apoptosis: A novel activity associated with the cyclin-dependent kinase inhibitor p27. Can. Res..

[34] Hou Y (2019). Design, synthesis and biological evaluation of novel 7-amino-[1,2,4]triazolo[4,3-f]pteridinone, and 7-aminotetrazolo[1,5-f]pteridinone derivative as potent antitumor agents. Eur. J. Med. Chem..

[35] Offiong OE, Martelli S (1997). Stereochemistry and antitumour activity of platinum metal complexes of 2-acetylpyridine thiosemicarbazones. Transit. Met. Chem..

[36] Hall IH (1993). The cytotoxicity of heterocyclic thiosemicarbazones and their metal complexes on human and murine tissue culture cells. Anticancer Drugs.

[37] Alcaraz R (2020). Thiosemicarbazone-metal complexes exhibiting cytotoxicity in colon cancer cell lines through oxidative stress. J. Inorg. Biochem..

