# Supplementary material for: Enhanced anticancer potency with reduced nephrotoxicity of newly synthesized platin-based complexes compared with cisplatin
Source: Sci Rep. 2022 May 18;12:8316. doi: 10.1038/s41598-022-11904-3 (PMC9117324; doi:10.1038/s41598-022-11904-3)
Supplement: Supplementary file 1 — Supplementary Information. [file 41598_2022_11904_MOESM1_ESM.docx]

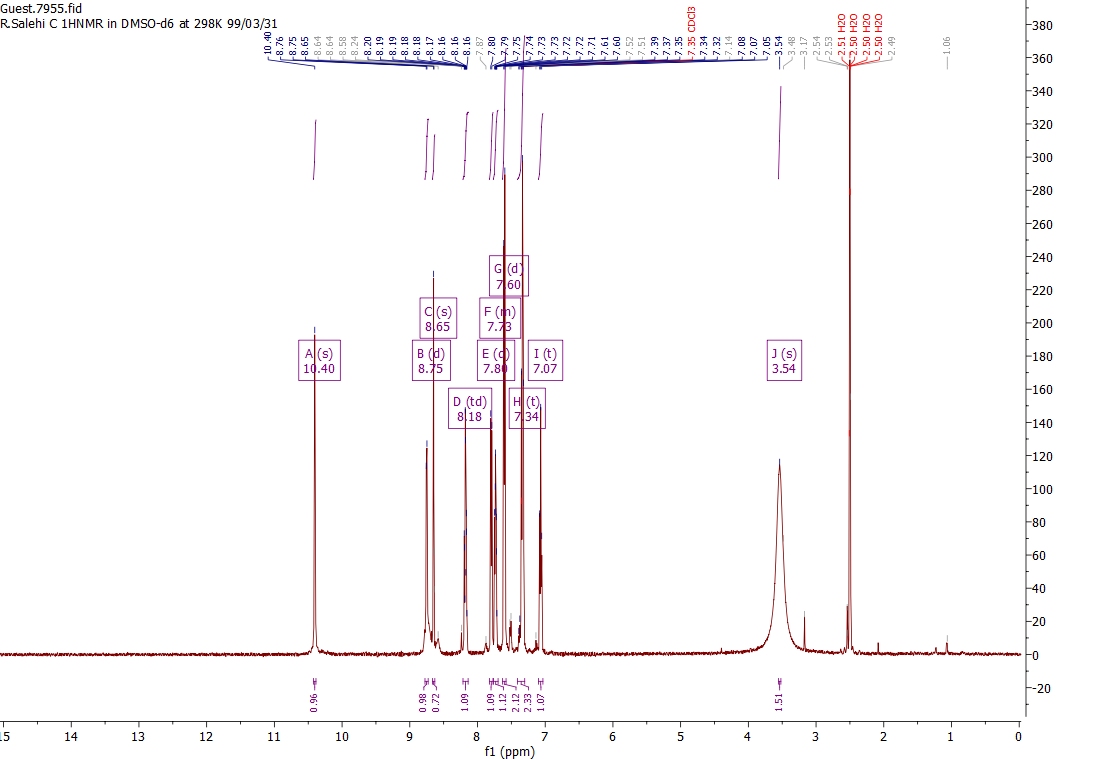


Figure S1. 1HNMR spectrum of Pt(TSC)Cl complex

Figure S2. FT-IR spectrum of Pt(TSC)Cl complex


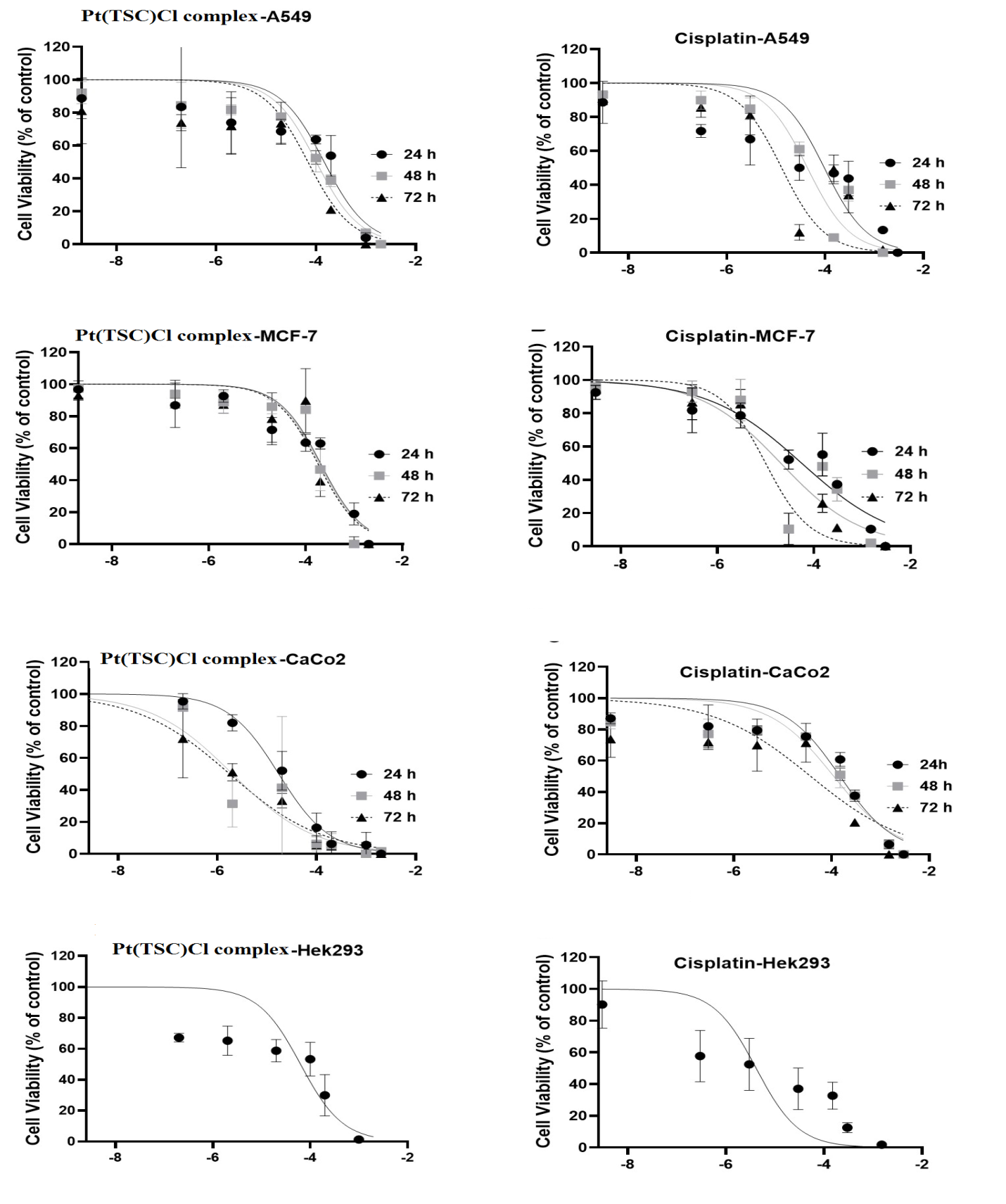


Figure S3. Effect of Pt(TSC)Cl complex and cisplatin on cancer cell growth. A549, MCF-7 and Caco-2 (0.02–2000 µmol/L) cancer cells were treated with Pt(TSC)Cl complex and cisplatin in cell culture medium for 24, 48 and 72 h. Hek293 normal cells were treated with Pt(TSC)Cl complex and cisplatin in cell culture medium for 24 h. Data are expressed as the mean ± SEM of three dependent experiments (n =3).


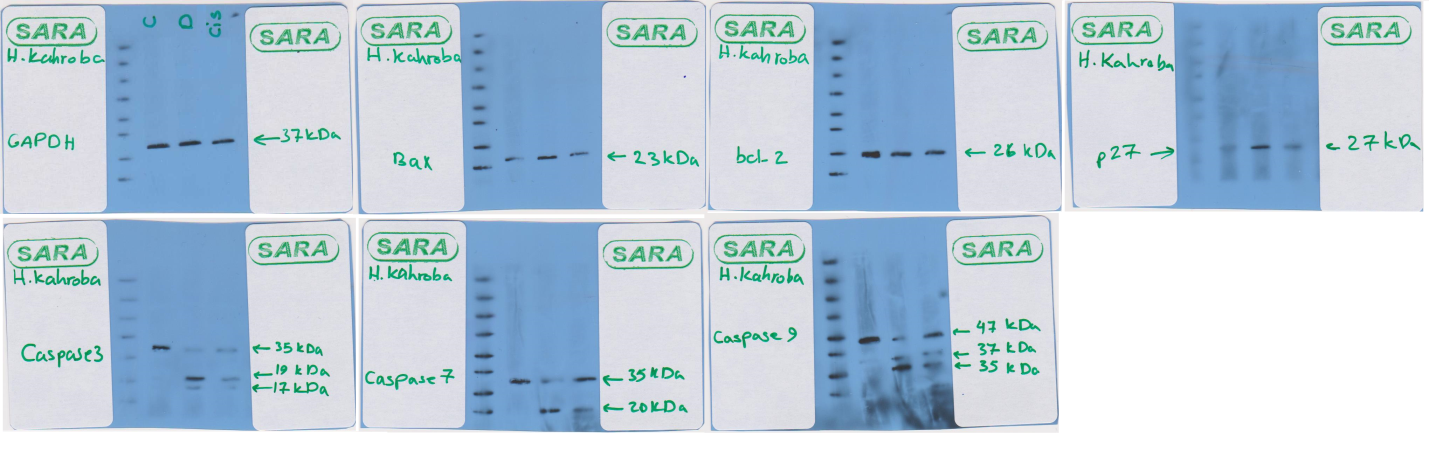


Figure S4: Full-length gels and blots of proteins expression obtained from western blotting of the CaCo2 cells treated with IC50 value of Cisplatin and Pt(TSC)Cl complex. Un-treated cells were considered as the control group. Proteins: GAPDH as internal control, Bcl-2, Bax, p27, pro-Caspase-3, Cleaved-Caspase-3, pro-Caspase-7, Cleaved-Caspase-7, pro-Caspase-9, and Cleaved-Caspase-9.
